# Human nasal rhinosporidiosis: a case report from Malawi

**DOI:** 10.4314/pamj.v9i1.71203

**Published:** 2011-07-18

**Authors:** Sefu Uledi, Ayubu Fauzia

**Affiliations:** 1 Department of Surgery, Mzuzu Central Hospital, Malawi; 2 Department of Obstetrics and gynaecology, Mzuzu Central Hospital, Malawi

**Keywords:** Rhinosporidiosis, polyps, nose, Malawi

## Abstract

Rhinosporidiosis is a rare chronic granulomatous disease, characterised by polypous lesions of the mucous membrane. Commonly affects the mucous membrane of the naso-pharynx, conjunctiva and palate. Its causative agent is *Rhinosporidium seeberi*. The disease is more prevalent in the Indian subcontinent, but remains quite rare in our environment. We hereby present a case description of a 70 year old native Malawian male with a polypoid nasal rhinosporidiosis. Patient presented with long standing history of nasal obstruction and intermittent epistaxis for three years. Diagnosis was confirmed by histopathological examination and he was successfully treated by complete surgical excision. This was a very unusual cause of nasal masses in our setting. Nasal rhinosporidioss lesions may largely mimic other ordinary nasal polyps, it is crucial therefore for clinicians in our region to consider rhinosporidiosis as a differential diagnosis when assessing patients presenting with nasal swellings.

## Introduction

Rhinosporidiosis is a rare infective granulomatous disease caused by *Rhinosporidium seeberi*. The causative pathogen is widely deemed to be a fungus though hitherto its precise taxonomy remains indistinct. The disease predominantly affects mucosal lining of the naso-pharynx, conjunctiva, palate and urethra. Lesions involving other regions of the body like brain, trachea, ear, skin and subcutaneous tissues have been reported but are rather uncommon [1].

This disorder exhibits no racial predilection although its pattern displays male gender preponderance. Besides humans the disease has been reported in other animals such as horses and bovines [2–5]. Nasal rhinosporidiosis is endemic in India, Sri Lanka, and Bangladesh with sporadic cases reported from other parts of the world such as Argentina, Brazil, Italy, Iran, Tanzania, Nigeria, and Uganda [1–6].

Few cases of conjuctival rhinosporidiosis have been reported from Malawi [7]. Never the less it remains exceedingly infrequent disease entity in our region; to the author's knowledge by far there is no documented report on human nasal rhinosporidiosis from Malawi.

This report therefore is aimed at documenting a case of 70 year old native Malawian male with a polypoid nasal rhinosporidiosis. Also caution clinicians in our region to have high index of suspicion when evaluating patients with nasal lumps. It should be noted that, contrary to ordinary nasal polyps, rhinosporidiosis lesions are associated with high rate of recurrence if surgical extirpation is not executed with extra prudence.

## Case report

A 70-years old male who was referred to our hospital with long standing history of right-sided nasal obstruction and gradual but progressively mounting right nasal cavity growth for three years. Signed written informed consent for publication of this report was obtained from the patient.

Associated with nasal growth patient gave history of occasional epistaxis, intermittent post-nasal drip and punctuated spells of nasal itching. He reported no history of significant constitutional symptoms. Patient has been a peasant paddy cultivator for the past forty five years. He reports no history of similar illness in the family.

**Figure 1:**
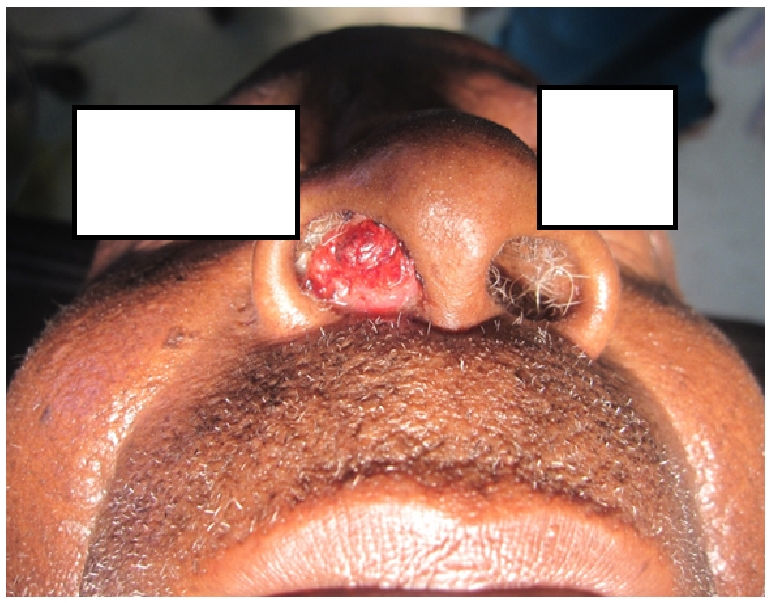
Polypoidal nasal mass which had grown up gradually and stuffed the right nasal cavity

On examination, the significant findings were on local examination, however on general examination; we saw an elderly man in good nutritional status, not pale, not dyspneic and had no any conjuctival growth. Locally, there was a friable slightly mobile polypoidal mass filling the right nasal cavity. The mass was erythematous, non-tender and bleeds easily on contact. It was about 4cm in diameter with a short pedicle arising from the lateral aspect of the left inferior turbinate (Figure 1). The contra lateral nasal cavity, naso-pharynx and palate were normal. No enlargement of local-regional lymph nodes observed. Other systemic examinations were unremarkable.

**Figure 2:**
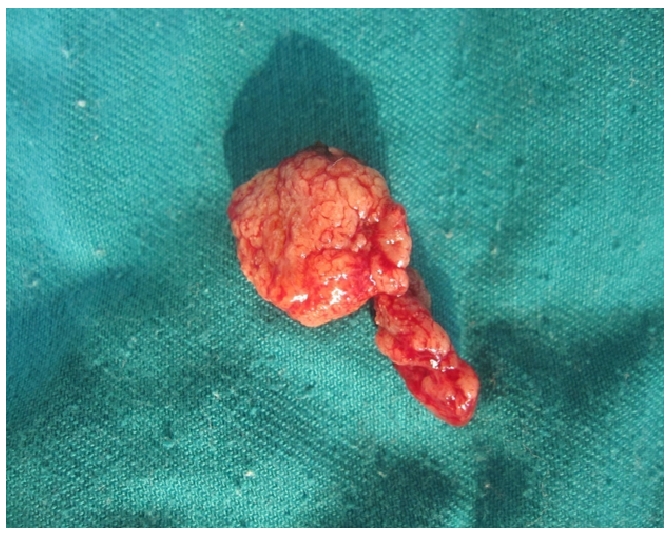
An intact pedunculated nasal mass was successfully excised. It was friable in consistency and weighed 17.4g

Work-up of the patient comprised of biochemical and haematological profiles as well as incisional biopsy from the lesion. Biochemical and haematological parameters were essentially normal. However, histopatholgical examination of the tissue showed sub mucosal globular cysts containing spores (sporangia) in different stages of development; these findings were pathognomonic of rhinosporidiosis. Henceforth, a final diagnosis of rhinosporidiosis was embraced and the patient was planed for a definitive surgery whereby the nasal lump was meticulously and completely excised, coupled with electrical cauterisation of the lesion's base. Gross examination of the resected lesion depicted an intact polypoidal mass with a body and stalk like appearance. Measured a maximum diameter of 4 cm and weighed 17.4 grams. It was soft and friable in consistency (Figure 2).

Generally, the patient did remarkably well postoperatively and he had uneventful convalescence period. Follow-up visit six months later revealed no signs of recurrence.

## Discussion

Rhinosporidiosis has existed since ancient times. Initial account of this disease entity was made over a century ago in Latin America. Though it appears to occur universally, rhinosporidiosis remains largely endemic in Indian subcontinent [1–7]. The mode of transmission to humans is not clearly understood, however most researchers believes that direct contact with spores through, dust, soil or prolonged exposure to stagnant water are among the major risk factors for the infection acquisition [3,4,8]. We strongly believe that being a paddy grower for over forty years our patient had a very high risk of contracting such infection. Usually patient presents with history of gradual nasal growth, occasional epistaxis, nasal itching, sneezing, and at times post-nasal dripping [1–8]

Clinically nasal rhinosporidiosis is characterised by development of single pedunculated polyp, multiple sessile polypoid tumours or a combination of both. Contrary to ordinary polyps which often arise from the middle turbinate, rhinosporidiosis frequently involve mucosal lining of the naso-pharynx, anterior nares, inferior turbinate, septum or nasal floor. Therefore, nasal polyps originating from these locations should always be treated with high index of suspicion [9].

Apart from history and clinical findings, histopathology of the biopsied tissues from the lesion is mandatory for definitive diagnosis of rhinosporidiosis; typical feature being identification of the pathogen in its diverse stages of development [1–8]. Surgical excision remains the mainstay of treatment for rhinosporidiosis lesions. Wide, complete and meticulous excision of the polyp followed by thorough electro-cautery of the lesion's base is recommended [1–9]. It is hypothesized that cauterisation of the lesion's base may abate recurrence resulting from spillage of endospores on the adjacent mucosa [1–5].

Besides surgery, a variety of adjuvant medical therapies have been tried in treatment of rhinosporidiosis. These include drugs like griseofluvin, amphoterecin B and dapsone (4, 4-diaminodiphenyl sulphone). However, by far there has been no tangible success with medical therapy [2-5,8].

## Conclusion

Nasal rhinosporidiosis remains a seldom disease entity in our environment. However, with emanating reports of sporadic cases in our region, it is consequently imperative for clinicians in our setting to consider rhinosporidiosis as a differential diagnosis when evaluating patients presenting with nasal growth.
